# Relationship Between Ultrasound Features and Ki-67 Labeling Index of Soft Tissue Sarcoma

**DOI:** 10.3389/fonc.2021.687878

**Published:** 2021-06-28

**Authors:** Pingping Wang, Hai Li, Yu Hu, Xiaojing Peng, Xinhua Ye, Di Xu, Ao Li

**Affiliations:** ^1^ Department of Ultrasound, The First Affiliated Hospital of Nanjing Medical University, Nanjing, China; ^2^ Department of Pathology, The First Affiliated Hospital of Nanjing Medical University, Nanjing, China

**Keywords:** ultrasonography, Ki-67, soft tissue sarcoma, musculoskeletal, immunohistochemistry

## Abstract

**Objectives:**

To explore the relationship between ultrasound (US) features and Ki-67 labeling index (LI) of soft tissue sarcoma (STS).

**Methods:**

Forty-six patients with 47 STS lesions, between September 2014 and April 2020, were enrolled in the study. Point-biserial correlation analysis and Spearman’s correlation analysis were utilized to examining the relationship between the US features and the Ki-67 LI of STS. The differences of US features between high and low Ki-67 proliferation groups were statistically analyzed by independent t test, Wilcoxon rank-sum test, and Fisher’s exact test. The optimal cut-off points of US features revealing significant differences were estimated by the maximum Youden index.

**Results:**

A moderate correlation between the vascular density grade and the Ki-67 LI (ρ = 0.409, P = 0.004) was found in this study. In addition, other ultrasound features were irrelevant to the Ki-67 LI. The cut-off for differentiating low- and high-proliferation groups was grade II according to the best Youden index. The area under receiver operating characteristic (ROC) curve was 0.74 (p = 0.011) with a sensitivity of 60.6% and specificity of 78.6%.

**Conclusions:**

Only the vascular density grade of STS had a weak positive correlation with Ki-67 LI, and might be capable of predicting the proliferation of STS. Other ultrasonographic features of STS such as shape and tumor margin have no correlation with Ki-67 LI.

## Introduction

Soft tissue sarcomas (STS) account for 1% of all malignant tumors, which exhibit all malignant tumor behaviors and have complex histological subtypes (>50 types) (1). Appropriate individualized treatment can prolong the survival rate of STS patients and improve their life quality. Early comprehensive evaluation of the biological behavior of STS is crucial for clinical treatment selection and thus become a clinically challenge (2).

Ki-67, a nuclear protein related to cell proliferation ability, was firstly described in 1983 (3). In a variety of tumors (such as breast cancer, colorectal cancer, lung cancer), the effectivity of Ki-67 as a prognostic marker has been proven (4–7). Hoos et al. (8) carried out a retrospective study on 47 patients with STS, which demonstrated that high Ki-67 proliferation index could be an important factor in the prognosis of high-risk STS patients. In a prospective study, Tanaka et al. (2) proved that Ki-67 classification system was more effective and repeatable than the traditional French Federation of Cancer Centers Sarcoma Group (FNCLCC) system.

Pathological immunohistochemistry (IHC) is often used to detect Ki-67 labeling index (LI), which is an invasive method with time delay. Therefore, researchers began to explore a simple and non-invasive method to evaluate Ki-67 LI and made some preliminary breakthroughs. For instance, the preoperative magnetic resonance imaging (MRI) parameters of small invasive hepatocellular carcinoma have been proved to be related to Ki-67 LI (9). In the study of Chen et al. (10), the spectral computed tomography (CT) imaging parameters in lung adenocarcinoma were moderately positively correlated with Ki-67 LI. In addition, Liu et al. (11) developed a multi-parameter MRI radiology model based on deep learning to improve the ability to predict the Ki-67 status of preoperative breast cancer.

Ultrasound (US) examination has the advantages of convenience, cheapness, real-time, and few contraindications (12). It can not only provide tumor size, shape, edge, internal structure, and other information, but also detect tumor blood flow with color Doppler US (12, 13). To our knowledge, no studies have researched the correlation between Ki-67 expression and the US characteristics of STS. In this study we investigated their relationship and whether US could be used as a non-invasive tool to evaluate the cell proliferation of STS.

## Materials and Methods

### Patients

**Figure 1:**
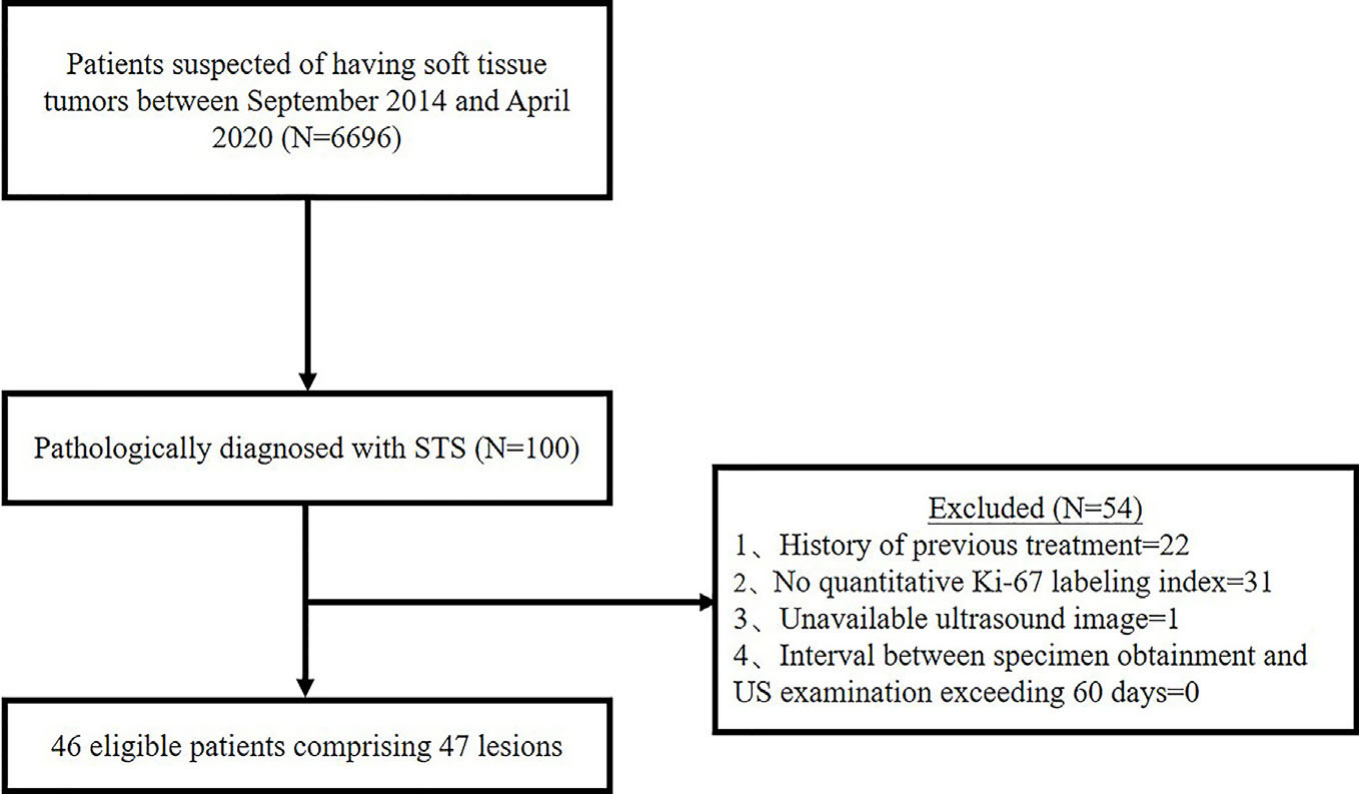
Flowchart of study population selection. (STS, soft tissue sarcoma; N, number).

Between September 2014 and April 2020, 6,696 patients had US scan for suspected soft tissue tumor and 100 patients were diagnosed as STS by pathology. Thenceforth, 54 cases were further excluded based on the following exclusion criteria: History of previous treatment (n = 22); No quantitative Ki-67 LI (n = 31); Unavailable US images (n = 1); Interval between specimen obtainment and US examination exceeding 60 days (n = 0). In the present study, we focused on soft tissue sarcoma of the musculoskeletal system. Gynecological or retroperitoneal tumors was not included. Ultimately, 46 patients comprising 47 lesions were enrolled in the study (**Figure 1**). This retrospective study was approval by the ethics committee of our hospital, and the requirement for informed consent was waived for all participants.

### Ultrasound Examination

US images were acquired using Logiq E9 (GE Healthcare, Milwaukee, WI, USA) US 54 machine with a 6–15 MHz liner probe and a 1–6 MHz convex probe, and an ACUSON S3000 Ultrasound System with a 9L4 probe (4–9 MHz) (Siemens Medical Solution, Mountain View, CA, USA). Patients were in a position where the lesions could be completely exposed. In order to optimize the color blood flow display, ultrasound gel and light pressure need to be applied in the lesion area. The gray-scale US was performed to observe and record the shape, size, location, boundary, internal echo, surrounding tissue relationship and capsule of the mass in detail. Subsequently, color doppler flow imaging (CDFI) was used to detect the vascular density and distribution of the mass. Spectral analysis and elastography were not routinely performed.

### Ultrasound Features Extraction

**Figure 2:**
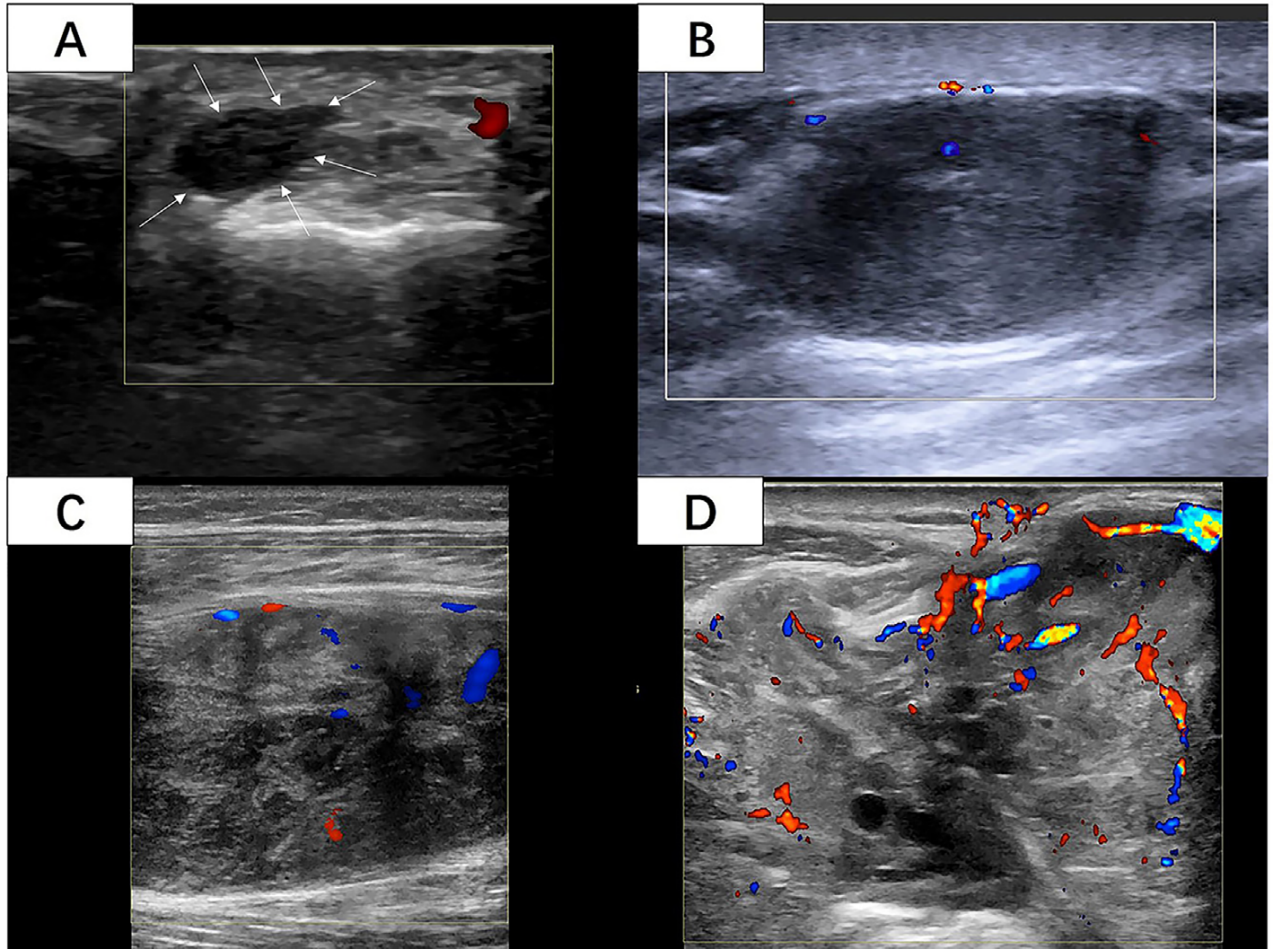
**(A)** A 42-year-old woman with 6cm lesion with vascular density grade 0 with histological diagnosis of synovial sarcoma with Ki-67 LI of 5%. (White arrow: The margin of the lesion) **(B)** A 50-year-old woman with 27cm lesion with vascular density grade I with histological diagnosis of leiomyosarcoma with Ki-67 LI of 10%. **(C)** A 33-year-old woman with 73cm lesion with vascular density grade II with histological diagnosis of fibrosarcoma with Ki-67 LI of 10%. **(D)** A 70-year-old man with 86cm lesion with vascular density grade III with histological diagnosis of synovial sarcoma with Ki-67 LI of 60%. (Images were obtained from four different lesions included in our study.

Two radiologists who had 5 and 10 years of experience in musculoskeletal US independently reviewed the images from the Picture Archiving and Communication System (PACS) of our hospital and recorded all the US features of the STS blinded to patient information and histological diagnosis. Over five static US images including at least two CDFI images were assessed for each lesion. The following US features were evaluated and recorded: (1) The maximum diameter of the mass was the average value after multiple measurements. (2) The shape consisted of regular (round, oval) and irregular. (3) The tumor margin was assessed as clear (well-defined: capsule like; clear-cut and thin) or unclear (ill-defined: uncertain boundary with adjacent normal tissue, or certain boundary with irregular margin and wider transitional zone); (4) The internal echo included three types in contrast to adjacent normal tissue: hypoechoic, hyperechoic, and isoechoic. (5) Internal texture was classified as homogeneous and inhomogeneous. (6) If the internal component was more than 50% cystic, it was mainly cystic, otherwise it was mainly solid. Internal calcifications were assessed as positive or negative. (7) The vascular density was assessed as four grades by the criteria of Adler et al. (14) (**Figure 2**): Grade 0 showed no blood flow signal in the mass; Grade I showed a small amount of blood flow: one to two punctate or rod like vessels could be seen in the mass; Grade II showed moderate blood flow: three to four punctate vessels or a long strip shape blood vessel penetrated into the lesion with a length close to or beyond the radius of the mass; Grade III showed multiple blood flow: more than five punctate vessels or two long strip shape vessels in the mass (8). The vascular distribution was identified as none, peripheral, internal, and mixed.

### Histopathology and Immunohistochemistry

The immunohistochemistry (IHC) specimens of the included lesions, 40 cases were from surgery and 7 from biopsy. Formalin-fixed and paraffin-embedded samples were stained with hematoxylin and eosin and analyzed by IHC using Ki-67 antibodies (1:200, Dako). An experienced sarcoma pathologist reviewed the IHC slides. The Ki-67 LI was recorded as a percentage of Ki-67 positive tumor cells. According to the IHC staining, the included lesions was divided into two groups (8): The Ki-67 index is considered to be high if >=30% tumor cells showed stained nuclei and low if <30%.

### Statistical Analysis

We assessed the correlation between US features and Ki-67 LI (high/low): point-biserial correlation analysis for continuous variables; Spearman correlation analysis for categorical variables. When the absolute value of correlation coefficient was 0–0.2, no or very weak correlation was indicated; 0.2–0.4, low; 0.4–0.6, moderate; 0.6–0.8, high; 0.8–1, very strong. The differences in US features were analyzed between high and low Ki-67 proliferation groups. The independent t test or Wilcoxon rank-sum test was used for continuous and ordinal categorical variables. The chi-squared test or Fisher’s exact test was utilized for categorical variables. According to features revealing significant differences, we computed receiver operation characteristic (ROC) curves and calculated the area under the curve, respectively. The optimal cut-off points were estimated by the maximum Youden index (sensitivity+specificity-1). Consistency of the observers was evaluated using intraclass correlation (ICC) and kappa for continuous and categorical variables, respectively. When ICC/kappa was 0–0.4, consistency was considered poor; 0.4–0.59, general; 0.6–0.74, good; 0.75–1, excellent (15). All statistical analyses were performed in the IBM SPSS Statistics 25 (IBM SPSS, Turkey) program. If P < 0.05, it was considered statistically significant.

## Result

**Figure 3:**
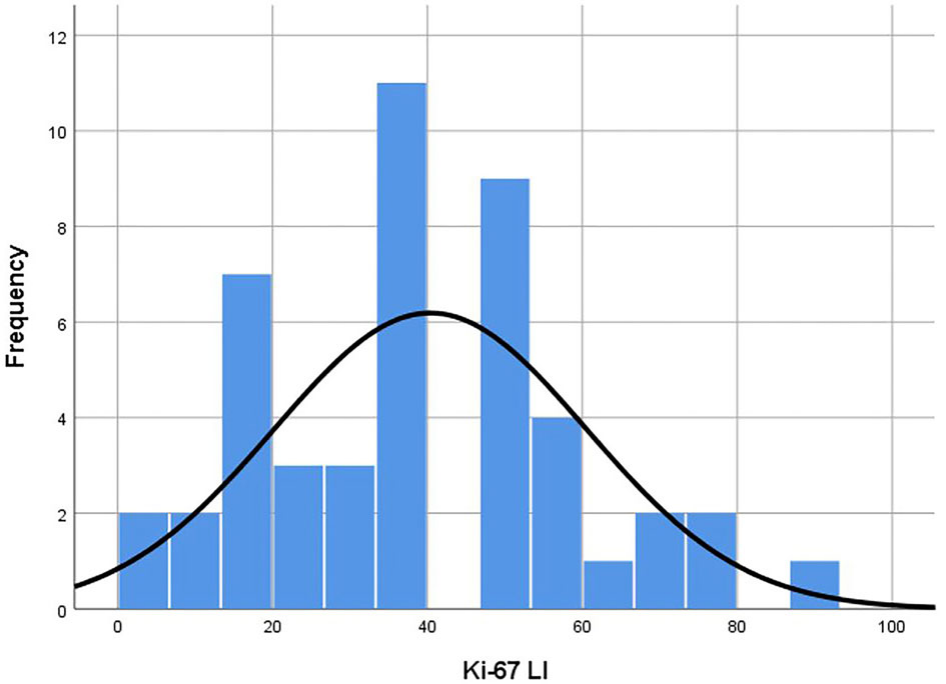
Histogram of Ki-67 LI distribution of the lesions. Distribution was in accordance with the Kolmogorov-Smirnov test (p = 0.20). Median was 40.00 and standard deviation was 20.20. LI, labeling index.

The average age of 46 patients was 58.2 (range 12–92) years old. There were 47 lesions, 1 in the head and neck, 30 in the extremities, and 16 in the trunk. The distribution range of Ki-67 LI was 2–90% (**Figure 3**). Time interval between ultrasound examination and pathological diagnosis was 2–51 days. Of the 47 lesions, 33 were in the high proliferation group and 14 were in the low group (**Table 1**).

**Table 1:** Detailed histological diagnoses in the low- and high-proliferation groups.

	High-proliferation group (N = 33)	Low-proliferation group (N = 14)
**Final pathological diagnosis**	Undifferentiated pleomorphic sarcoma = 7	Dermatofibrosarcoma protuberans =1
	Myxofibrosarcoma = 7	Alveolar soft part sarcoma =1
	Synovial sarcoma = 4	Synovial sarcoma=3
	Ewing’s sarcoma = 2	Myxoid liposarcoma = 5
	Clear cell sarcoma = 2	Clear cell chondrosarcoma =1
	Liposarcoma = 5	Liposarcoma = 2
	Leiomyosarcoma = 1	Leiomyosarcoma = 1
	Embryonal rhabdomyosarcoma = 1	
	Fibrosarcoma = 3	
	Small round cell undifferentiated sarcoma = 1	

N, number.

**Figure 4:**
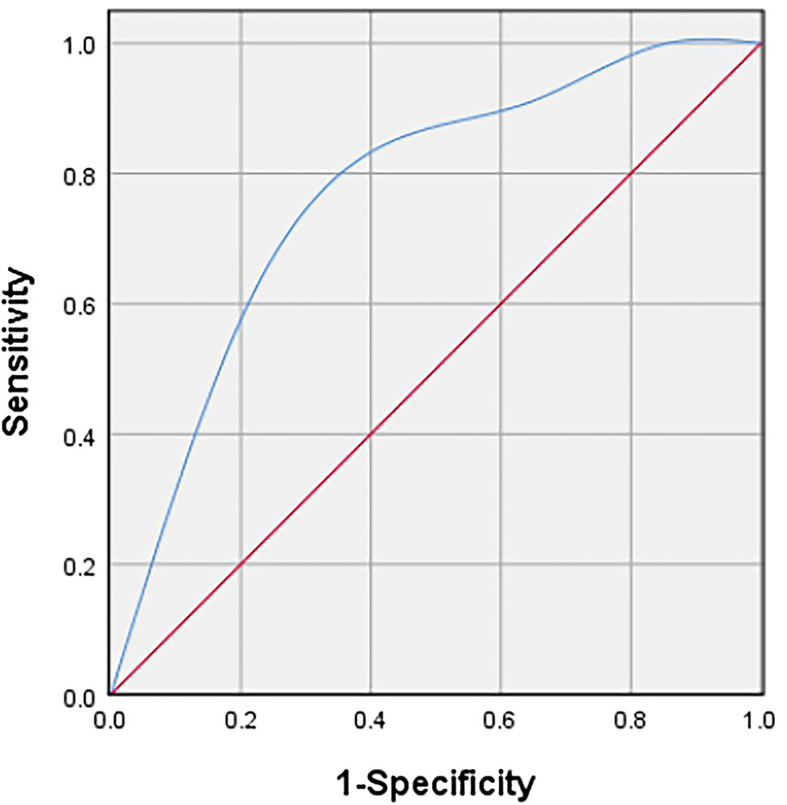
Receiver operating characteristic (ROC) curve to determine the cut-off value of the vascular density grade with Adler criteria in discriminating between high and low proliferation groups.

**Table 2** showed the distribution of general characteristics and ultrasound features of 47 lesions in the high- and low-proliferation groups. Additionally, the correlation of these parameters with KI-67 status and whether these parameters were statistically different in the high- and low-proliferation groups were also included in the table. As shown in **Table 2**, there was a moderate correlation between the vascular density grade of STS and the proliferation level (high/low) of Ki-67 with a correlation coefficient of 0.409 (P = 0.004). At the same time, there was a significant difference in the grade of vascular density between high- and low-proliferation groups (P = 0.005). The most appropriate “cut-off” of vascular density to evaluate the proliferation ability was between grade II and III. This meant that in clinical practice, if the ultrasound results of STS show that the vascular density is grade III, it may have high Ki-67 LI with exuberant cell proliferation ability; if the vascular density of STS is grade 0/I/II, it may have low Ki-67 LI with meagre cell proliferation ability. The sensitivity and specificity for differentiating low- and high-proliferation groups was 60.6 and 78.6%, with an area under the ROC curve of 0.74 (p = 0.011) (**Figure 4**). Moreover, the diameterMAX and depth of the STS demonstrated correlation respectively (ρ = 0.28, 0.25) between high and low groups that nearly met statistical significance (p = 0.06 and 0.09). However, shape, margin, internal echo, internal texture, internal component, calibrations, and the blood flow distribution had no correlation with the Ki-67 LI, and there was no significant difference between the high and low groups.

**Table 2:** Characteristics of soft tissue sarcomas.

Features		Number	High proliferation group (N = 33)	Low proliferation group (N = 14)	Coefficient ofcorrelation and P value	P value of diversity
Gender	Male	29	20 (69.0%)	9 (31.0%)	-0.04 (P=0.82)	P=0.81
	Female	18	13 (72.2%)	5 (27.8%)		
Age	/	47	58.21±17.91	58.29±17.87	-0.002 (P=0.99)	P=0.99
Position	Extremities	30	21 (70.0%)	9 (30.0%)	-0.016 (P=0.91)	P=1.00
	trunk	16	11 (68.8%)	5 (31.2%)		
	Head and neck	1	1 (100.0%)	0 (0.0%)		
Tissue layer	Superficial fascia	15	8 (53.3%)	7 (46.7%)	-0.25 (P=0.09)	P=0.10
	Deep fascia	32	25 (78.1%)	7 (21.9%)		
Diameter _MAX_	/	47	67.21±42.02	44.00±22.91	0.28 (P=0.06)	P=0.06
Shape	Regular	20	14 (70.0%)	7 (30.0%)	-0.004 (P=0.98)	P=0.98
	Irregular	27	19 (70.4%)	8 (29.6%)		
Margin	Clear	30	22 (73.3%)	8 (26.7%)	-0.09 (P=0.55)	P=0.53
	Unclear	17	11 (64.7%)	6 (35.3%)		
Internal echo	Hypoechoic	44	30 (68.2%)	14 (31.8%)	-0.17 (P=0.25)	P=1.00
	Isoechoic	1	1 (100%)	0 (0%)		
	Hyperechoic	2	2 (100%)	0 (0%)		
Internal texture	Homogeneous	5	2 (40%)	3 (60%)	-0.23 (P=0.12)	P=0.15
	Inhomogeneous	42	31 (73.8%)	11 (26.2%)		
Internal component	Mainly cystic	1	1 (100%)	0 (0%)	-0.10 (P=0.52)	P=1.00
	Mainly solid	46	32 (69.6%)	14 (30.4%)		
Calcification	Positive	6	4 (66.7%)	2 (33.3%)	-0.03 (P=0.84)	P=1.00
	Negative	41	29 (70.7%)	12 (29.3%)		
Grade of vascular density**	Grade 0	2	0 (0%)	2 (100%)	0.409 (P=0.004)	P=0.005
	Grade I	6	3 (50%)	3 (50%)		
	Grade II	16	10 (62.5%)	6 (37.5%)		
	Grade III	23	20 (87%)	3 (13%)		
Vascular distribution	None	2	0 (0%)	2 (100%)	-0.1 (P=0.51)	P=0.18
	Peripheral	1	1 (100%)	0 (0%)		
	Internal	5	4 (80%)	1 (20%)		
	Mixed	39	28 (71.8%)	11 (28.2%)		

**P ≤ 0.005; N, number.

The ICC and kappa values showed excellent agreement between observers (**Table 3**).

**Table 3:** Consistency of the observers with regard to US features of soft tissue sarcomas.

Features	ICC/Kappa
Diameter_MAX_	0.98
Shape	0.83
Margin	0.80
Internal echo	0.81
Internal texture	0.74
Internal component	0.84
Calcification	0.88
Grade of vascular density	0.86
Vascular distribution	0.91

ICC, intraclass correlation coefficient.

## Discussion

At present, the correlation between the imaging manifestations of STS and its prognosis has gradually attracted attention and research, providing more auxiliary suggestions for clinical treatment. Our study is the first to explore the correlation between US features and Ki-67 LI of musculoskeletal soft tissue sarcoma. The final result illustrated that there was a positive correlation between the STS vascular density grade and Ki-67 LI (ρ = 0.409, P = 0.004). The sensitivity and specificity to distinguish between low-proliferation and high-proliferation groups were 60.6 and 78.6%, respectively. And the area under ROC curve was 0.74 (p = 0.011). This indicates that US is not only cheap, convenient, real-time and easy-to-follow up, but also has potential in predicting the cell proliferation of STS.

Higher Ki-67 LI means thriving proliferation ability of tumor cells, which can be manifested by the increase of cell number and cell volume. Simultaneously, too rapid tumor proliferation causes extensive pathological angiogenesis, which is essential and integral for the growth of solid tumors (such as STS). In this process, vascular endothelial growth factor (VEGF) can effectively induce the formation of messy and fragile blood vessels and provide nutrients and oxygen for tumor. Nakopoulou et al. (16) found that VEGF was positively correlated with Ki-67 expression in breast carcinomas. In this study, we found that when the Ki-67 LI of STS was high, the blood vessel density was relatively increased. This was consistent with other types of tumors, such as breast cancer and multiple myeloma (17–19).

In addition to blood flow density, the diameterMAX and tissue layer (depth) showed a correlation with Ki-67 (ρ = 0.28, 0.25), which was very close to statistical significance (P = 0.06, 0.09). These two features are universally considered to be related to the prognosis of STS. Deeply located and greater than 5 cm in size are the risk factors of STS metastasis (20, 21). However, Soffer et al. found that although the combination of Ki-67 and tumor size of rhabdomyosarcoma had an association with lymph node proliferation, Ki-67 LI was not directly interrelated to tumor size (22). This appeared to be in line with our results. But it might be due to the small sample size of our study.

What’s more, there was no correlation between the Ki-67 LI and the internal echo, internal structure, internal component, margin, shape, or blood flow distribution type of the mass in our study. However, the correlation between these US features and Ki-67 LI was found in breast cancer and glioma. In intraoperative ultrasonography (IOUS), Baskan et al. (23) found that the pathogenicity of the solid parts, margins, and contours of glioma were related to the level of Ki-67. Zhang et al. (24) discovered that breast cancer with heterogeneous internal echo had stronger proliferative ability and poor prognosis. Compared with other studies, in addition to the differences in tumor types, the constituent cells of different subtypes of STS are diverse. In addition, excessive neovascularization can increase the central pressure of the tumor, which may lead to internal necrosis and liquefaction (25). All of these result in the disorder of tumor internal structure and complex components. In particular, the internal structure and echo are greatly affected by the tissue composition and cell density.

Recently, Lee et al. (26) found that the average apparent diffusion coefficient (ADCmean) on MRI was negatively correlated with Ki-67 LI of STS. Furthermore, correlation between the standard uptake value (SUV) of PET and a combination of cellularity and Ki-67 LI was already described (27). However, these studies had similar low sensitivity and specificity to our study, which might also be related to intricate subtypes and biological behaviors of STS mentioned above. It was worth noting that even though the best “cut-off” for the vascular density grade was level II in our study, its sensitivity was low. Which signified that 39.4% of STS would still escape diagnosis under this criterion. It can be fatal for patients. Therefore, it is undeniable that the vascular density grade of STS in US can only be utilized as an auxiliary method for clinicians in the diagnosis and follow-up process.

This study has the following limitations. First of all, it was a retrospective study. Bias in US examination and feature extraction might be occurred as the US machines and examiners were different. Secondly, some STS lesions were large. Although we evaluated the lesions from as many images as possible, the stored ultrasound images were only a cross-sectional view and might not represent the whole. Moreover, some cases using core needle biopsy specimens to evaluate Ki-67 LI was another shortcoming of our study. In addition, the histological classification of STS was complicated in general, but the sample size of STS included in this study was small and the pathological type was limited, which might lead to selection bias. Finally, in this study, we only used the conventional US features. Other US technologies, such as contrast-enhanced ultrasound and elastography, would be further studied in the future.

In conclusion, our study exhibited that the US features of STS had no significant correlation with Ki-67 LI, except for the moderate positive correlation of vascular density. Although its sensitivity and specificity are not satisfactory, as a non-invasive imaging method, US may help differentiate between STS with low and high proliferation potential and further studies are needed.

## Data Availability Statement

The raw data supporting the conclusions of this article will be made available by the authors, without undue reservation.

## Ethics Statement

The studies involving human participants were reviewed and approved by the ethics committee of the First Affiliated Hospital of Nanjing Medical University. Written informed consent to participate in this study was provided by the participants’ legal guardian/next of kin.

## Author Contributions

PW: Conceptualization, writing—original draft, software, and editing. HL: Writing—original draft, resources, and investigation. YH: Resources and investigation. XP: Resources and investigation. XY: Resources and investigation. DX: Conceptualization and project administration. AL: Data curation, conceptualization, and writing—review and editing. All authors contributed to the article and approved the submitted version.

## Funding

This work was supported by the Jiangsu Province Key Research & Development Plan (No. BE2018703) and the National Natural Science Foundation of China (No. 81401427).

## Conflict of Interest

The authors declare that the research was conducted in the absence of any commercial or financial relationships that could be construed as a potential conflict of interest.
